# (Pro)renin receptor is crucial for Wnt/β-catenin-dependent genesis of pancreatic ductal adenocarcinoma

**DOI:** 10.1038/srep08854

**Published:** 2015-03-09

**Authors:** Yuki Shibayama, Takayuki Fujimori, Genevieve Nguyen, Takuo Hirose, Kazuhito Totsune, Atsuhiro Ichihara, Kento Kitada, Daisuke Nakano, Hiroyuki Kobori, Masakazu Kohno, Tsutomu Masaki, Yasuyuki Suzuki, Shinichi Yachida, Akira Nishiyama

**Affiliations:** 1 Department of Pharmacology, Faculty of Medicine, Kagawa University, Kagawa 761-0793, Japan; 2 Department of Gastroenterology and Neurology, Faculty of Medicine, Kagawa University, Kagawa 761-0793, Japan; 3 Department of Cardiorenal and Cerebrovascular Medicine, Faculty of Medicine, Kagawa University, Kagawa 761-0793, Japan; 4 Department of Gastroenterological Surgery, Faculty of Medicine, Kagawa University, Kagawa 761-0793, Japan; 5 Center for Interdisciplinary Research in Biology (CIRB), Collège de France, Centre National de la Recherche Scientifique (CNRS) Unités Mixtes de Recherche (UMR) 7241, Institut National de la Santé et de la Recherche Médicale (INSERM) U1050, Paris 75005, France; 6 Department of Planning for Drug Development and Clinical Evaluation, Tohoku University Graduate School of Pharmaceutical Science and Medicine, Sendai 980-8577, Japan; 7 Department of Hypertension and Endocrinology, Tokyo Women's Medical University, Tokyo 162-8666, Japan; 8 Division of Refractory Cancer Research, National Cancer Center Research Institute, Tokyo 104-0045, Japan

## Abstract

Although Wnt/β-catenin signaling is known to be aberrantly activated in PDAC, mutations of *CTNNB1*, *APC* or other pathway components are rare in this tumor type, suggesting alternative mechanisms for Wnt/β-catenin activation. Recent studies have implicated the (pro)renin receptor ((P)RR) is related to the Wnt/β-catenin signaling pathway. We therefore investigated the possible role of (P)RR in pancreatic carcinogenesis. Plasma s(P)RR levels were significantly (*P* < 0.0001) higher in patients with PDAC than in healthy matched controls. We also identified aberrant expression of (P)RR in premalignant PanIN and PDAC lesions and all the PDAC cell lines examined. Inhibiting (P)RR with an siRNA attenuated activation of Wnt/β-catenin signaling pathway and reduced the proliferative ability of PDAC cells *in vitro* and the growth of engrafted tumors *in vivo*. Loss of (P)RR induced apoptosis of human PDAC cells. This is the first demonstration that (P)RR may be profoundly involved in ductal tumorigenesis in the pancreas.

Pancreatic ductal adenocarcinoma (PDAC) is a highly aggressive malignancy with few effective therapies and a very poor prognosis^1^,^2^. The disease is often diagnosed at an advanced stage, and less than 20% of patients are suitable for surgical resection^1^,^2^. Recently, several studies have estimated that the time from tumor initiation to metastatic dissemination is at least a decade by using mathematical analyses of tumor-DNA sequence data, suggesting that there is a window of opportunity for medical intervention before cancer spreads to distant organs^3^,^4^. Therefore, there is an urgent need to elucidate molecular mechanisms to facilitate early detection strategies and establishment of effective therapies.

The Wnt/β-catenin signaling pathway plays a critical role in multiple developmental events during embryogenesis, and is implicated in adult tissue homeostasis^5^. Recent studies have revealed important of inappropriate activation of the Wnt/β-catenin pathway to the progression of several cancers^6^,^7^ including PDAC^8^,^9^,^10^,^11^. Wnt binds to the Frizzled (Fz)/low-density lipoprotein receptor-related protein (LRP) complex at the cell surface, which leads to LRP phosphorylation and axin recruitment to the cell membrane^5^. This leads to the stabilization of β-catenin, which accumulates and translocates to the nucleus where it activates several transcription factors, such as lymphoid enhancer-binding factor 1/T-cell-specific transcription factor (LEF1/TCF). These latter factors promote cell proliferation by activation of target genes such as *CCND1*^5^. In genetically engineered mouse models of PDAC, cytoplasmic β-catenin expression was found to be markedly increased in pancreatic intraepithelial neoplasia (PanIN) lesions and tumors^12^,^13^. Conversely, constitutive activation of β-catenin is required for PanIN formation in mouse models^14^. *In vitro* studies also have shown that inhibition of Wnt/β-catenin signaling reduces cell proliferation and increases apoptosis in cultured PDAC cells^13^. However, Wnt/β-catenin signaling-related gene mutations are rare in PDAC tissues^11^,^15^,^16^,^17^,^18^,^19^ although several mutations of genes related to the activation of the Wnt/β-catenin signaling pathway have been demonstrated in other gastrointestinal cancers^20^. Thus, any common regulatory mechanism responsible for activation of the Wnt/β-catenin signaling pathway in PDAC is still largely a mystery.

The (pro)renin receptor ((P)RR) was discovered and successfully cloned by Nguyen *et al*.^21^. The human (P)RR complementary DNA encodes a 350-amino acid protein with a single transmembrane domain. The (P)RR gene is named *ATP6ap2/(P)RR*, which is located on the X chromosome (Xp11.4) in humans^21^,^22^. The (P)RR undergoes intracellular processing to generate three different molecular forms, a full-length integral transmembrane protein (full-length (P)RR), a soluble (P)RR (s(P)RR), and a truncated form composed of transmembrane and cytoplasmic domains associated with the vacuolar H^+^-proton adenosine triphosphatase (V-ATPase)^23^,^24^. Nguyen *et al*. also showed that (P)RR amplifies activation of the renin-angiotensin system targeted for renal and cardiovascular disorders. However, activation of (P)RR was shown to stimulate several intracellular signaling pathways independent of the renin-angiotensin system. Recent studies in *Xenopus*^25^ and *Drosophila*^26^,^27^ have indicated that (P)RR is involved in fundamental cellular functions related to the Wnt/β-catenin and Wnt/planar cell polarity signaling pathways. Nevertheless, the specific role of (P)RR in Wnt/β-catenin signaling in humans and especially in cancer remains to be investigated in detail.

In the present study, we aimed to elucidate the role of (P)RR in the pathogenesis of human PDAC focusing on PDAC-associated (P)RR expression and functions through the Wnt/β-catenin signaling pathway. We could demonstrate for the first time that (P)RR is profoundly involved in genesis of PDAC.

## Results

### Plasma s(P)RR levels in patients with PDAC are higher than those in healthy matched controls

**Figure 1:**
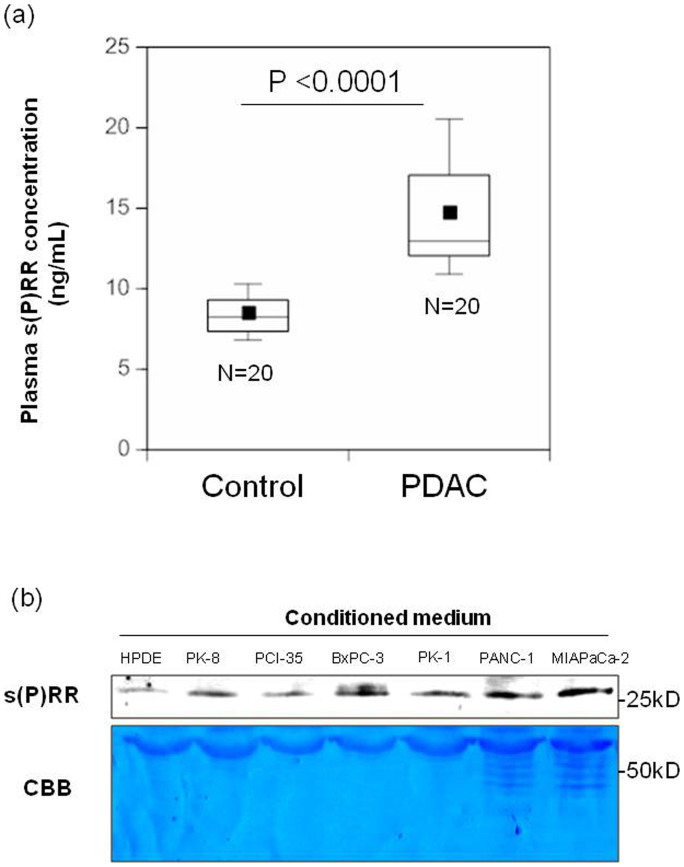
Plasma s(P)RR levels in patients with PDAC and amounts in the conditioned medium of human PDAC cell lines. (a) Plasma s(P)RR levels in 20 patients with PDAC and 20 healthy matched controls. Data are mean ± SD values. The boxes encompass the twenty-fifth through seventy-fifth percentiles of results. The lines through the middle of each box represent the medians. The maximum and minimum values within 1.5 × interquartile range (IQR) are shown as whisker caps. Average values are indicated by dots in the boxes. (b) s(P)RR in conditioned medium was assayed for HPDE cells and six different PDAC cell lines. Consistent results were observed when three experiments were repeated. Loading control was determined by CBB staining.

Fig. 1a shows plasma concentrations of s(P)RR in 20 patients with PDAC and 20 healthy controls. The information of age and gender of healthy control subjects was provided in supplementary Table S1. Supplementary Table S2 summarizes clinical characteristics of patients. Blood collection was performed during the same period in National Cancer Center, Tokyo Japan, and 20 potential controls were frequency-matched to the patients in eight age categories (50–54, 55–59, 60–64, 65–69, 70–74, 75–79, 80–84 and 85–89 years of age) and sex. As shown in Fig. 1a, plasma s(P)RR levels were significantly (*P* < 0.0001) higher in patients with PDAC (14.67 ± 3.64 ng/mL) than in healthy controls (8.45 ± 1.37 ng/mL).

s(P)RR is generated intracellularly through a furin cleavage mechanism and secreted^23^, enabling detection of full-length (P)RR and s(P)RR expression in human PDAC cells. Excretion of s(P)RR was investigated in six human PDAC cell lines (PK-8, PCI-35, BxPC-3, PK-1, PANC-1 and MIAPaCa-2) and human pancreatic duct epithelial (HPDE) cells. S(P)RR levels in the medium were higher in all six PDAC cell lines than in HPDE cells (Fig. 1b). Compared to HPNE (Human Pancreatic Nestin-Expressing) cells, s(P)RR expression of two human PDAC cell lines (BxPC-3 and PK-8) was also higher (Supplementary Fig. S1a).

### (P)RR is highly expressed in precursor and PDAC tissues and human PDAC cell lines

**Figure 2:**
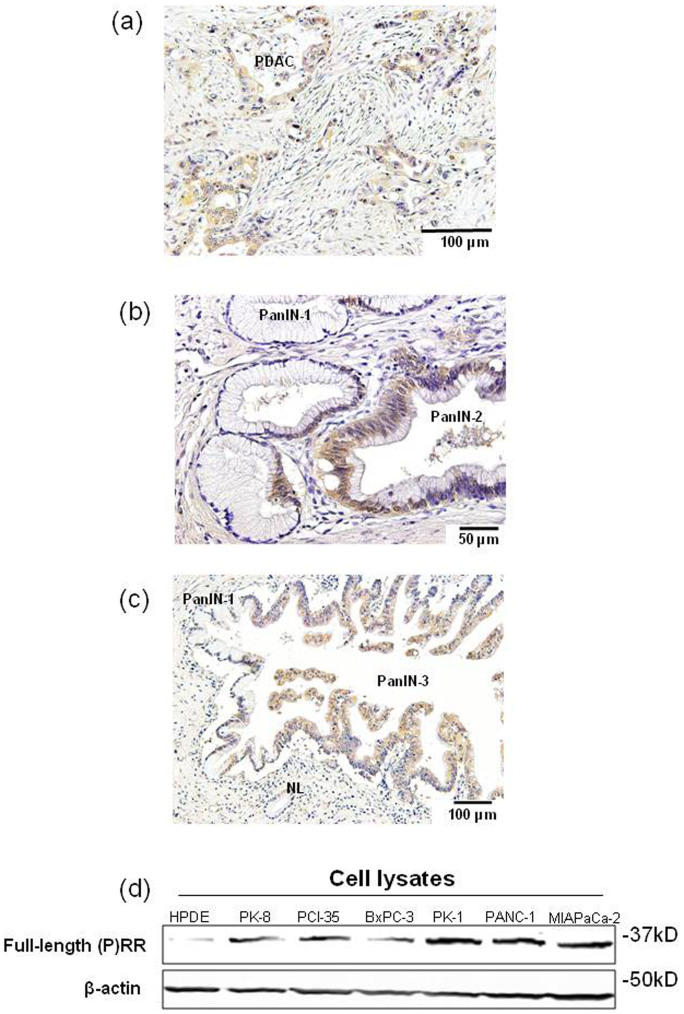
(P)RR is highly expressed in precursor and PDAC lesions and human PDAC cell lines. (a) Typical immunohistochemical labeling profiles of (P)RR in PDAC tissues. (b) (P)RR expression in PanIN-1 and PanIN-2 lesions in representative pancreatic tissue samples. The PanIN-2 lesions show strong (P)RR immunoreactivity in the cytoplasm, although the PanIN-1 lesions show only focal and faint (P)RR staining. (c) (P)RR expression in normal pancreatic duct (NL), and PanIN-1 and PanIN-3 lesions in representative pancreatic tissue samples. The PanIN-3 lesions show strong (P)RR immunoreactivity in the cytoplasm. (d) Protein expression of full-length (P)RR in cell lysates was measured in HPDE cells and six different PDAC cell lines. β-actin was used as a loading control. Consistent results were observed when three experiments were repeated.

Immunohistochemical analysis showed significant (P)RR expression in the cytoplasm of neoplastic epithelial cells in 21 of 22 samples of PDAC tissues obtained from patients who underwent resection (Fig. 2a). Supplementary Table S3 summarizes clinicopathologic features of these patients. We also examined whether (P)RR is highly expressed in pancreatic premalignant lesions called PanIN^28^. Interestingly, (P)RR was overexpressed in PanIN-2 and PanIN-3 lesions where atypical nuclei were observed (Fig. 2b, c). By contrast, (P)RR staining was very faint in normal pancreatic ducts and PanIN-1 lesions. These results suggest that aberrant (P)RR expression occurs from early stages of pancreatic tumorigenesis.

Full-length (P)RR expression in cell lysates was higher in all six PDAC cell lines than in HPDE cells (Fig. 2d).The consistent result was obtained even when we set HPNE cells as control (Supplementary Fig. S1b). These data supported the facts that (P)RR was overexpressed in human PDAC tissue samples.

### (P)RR is essential for activation of the Wnt/β-catenin signaling pathway in human PDAC cells

**Figure 3:**
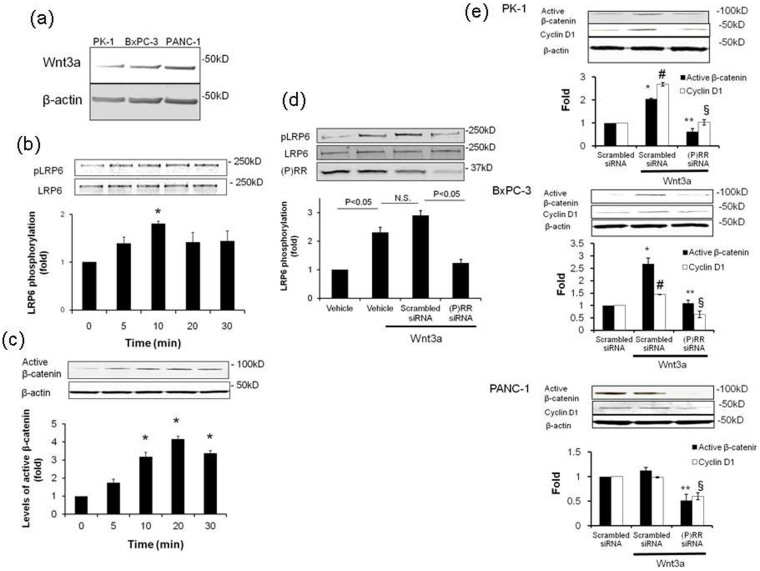
(P) RR is essential for activating the Wnt/β-catenin signaling pathway in human PDAC cell lines. (a) Representative image of Wnt3a expression among three human PDAC cell lines. Consistent results were observed when three experiments were repeated. (b) Wnt3a (150 ng/mL) significantly increased LRP6 activity in PK-1 cells (mean ± SEM, *n* = 3 for each). *P < 0.05 *vs.* vehicle-treated cells. Blotting with an anti-LRP6 antibody showed equal loading. (c) Wnt3a significantly increased active β-catenin expression in PK-1 cells (mean ± SEM, *n* = 3 for each). *P < 0.05 *vs.* vehicle-treated cells. β-actin was used as a loading control. (d) Effect of (P)RR siRNA on LRP6 activity in Wnt3a-treated PK-1 cells (mean ± SEM, *n* = 3 for each). Blotting with an anti-LRP6 antibody showed equal loading. (P)RR protein expression indicates efficient gene transfection. (e) Effect of (P)RR siRNA on active β-catenin and Cyclin D1 expression in PK-1, BxPC-3 and PANC-1 cells (mean ± SEM, *n* = 3 for each, *P < 0.05 *vs.* active β-catenin expression in scrambled siRNA-transfected cells; **P < 0.05 *vs.* active β-catenin expression in scrambled siRNA-transfected cells stimulated with Wnt3a; #*P* < 0.05 *vs.* CyclinD1 expression in scrambled siRNA-transfected cells; §*P* < 0.05 *vs.* Cyclin D1 expression of scrambled siRNA-transfected cells stimulated with Wnt3a). β-actin was used as a loading control.

We aimed to investigate whether (P)RR is a key factor for the canonical (β-catenin-dependent) Wnt signaling pathway in human PDAC cells. We examined the effect of Wnt3a, which is essentially involved in cell survival^29^,^30^, as previously reported in studies of the pancreas^13^,^31^. Endogenous Wnt3a expression was confirmed among three different PDAC cell lines. Wnt3a at basal conditions was highly expressed in PANC-1 cells (Fig. 3a). We focused on activation of Wnt3a-stimulated LRP6, which is a part of the Wnt receptor complex that also comprises (P)RR^25^, and on the non-phosphorylated form of β-catenin (“active” β-catenin) expression, which is thought to be responsible for mediating Wnt signaling in human PDAC cells^32^. In PK-1 cells, treatment with recombinant human Wnt3a (150 ng/mL) significantly enhanced phosphorylation of LRP6, a peak being reached after 10 min (Fig. 3b). Similarly, Wnt3a enhanced the expression of active β-catenin on and off from 10 min (Fig. 3c), suggesting that Wnt3a simultaneously induces phosphorylation of LRP6 and active β-catenin expression.

Treatment with (P)RR siRNA decreased in (P)RR expression in PK-1 cells (Fig. 3d). Wnt3a-induced LRP6 activation was dramatically reduced by (P)RR siRNA-treatment (Fig. 3d), compared with the cells treated with scrambled siRNA. Expression of active β-catenin and Cyclin D1 as translation products induced by Wnt target genes was significantly augmented by Wnt3a-stimulation in PK-1 and BxPC-3 cells (Fig. 3e). While increase in active β-catenin and Cyclin D1 expression in Wnt3a-stimulated PANC-1 cells was not observed, treatment with (P)RR siRNA significantly reduced levels of active β-catenin and Cyclin D1 in all three human PDAC cell lines (Fig. 3e).

Immunohistochemistry revealed that β-catenin was overexpressed in almost all human PDAC tissues (Supplementary Fig. S2a). The immunoreactivity was localized primarily in the membranes of normal ductal cells, but was evident in both the cytoplasm and nuclei in PDAC cells. While β-catenin immunoreactivity was faint in the membranes of PanIN-1 lesions, overexpression of β-catenin was observed in the cytoplasm or/and nuclei in PanIN-2 and PanIN-3 lesions (Supplementary Fig. S2b).

### (P)RR promotes human PDAC cell proliferation

**Figure 4:**
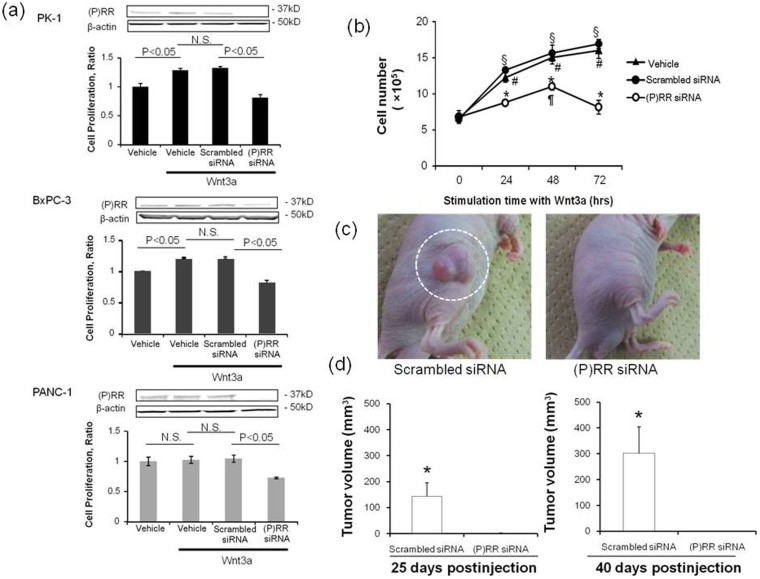
(P) RR is essential for Wnt3a-induced proliferation of human PDAC cells. (a) ***Top***: (P)RR expression and WST-1 proliferation assay in (P)RR siRNA-transfected PK-1 cells evaluated at 48 h after Wnt3a stimulation. ***Middle***: (P)RR expression and WST-1 proliferation assay in (P)RR siRNA-transfected BxPC-3 cells evaluated at 48 h after Wnt3a stimulation. ***Bottom***: (P)RR expression and WST-1 proliferation assay in (P)RR siRNA-transfected PANC-1cells evaluated at 48 h after Wnt3a stimulation. (b) Effect of (P)RR siRNA transfection on number of cells, as assessed by direct cell counting of Wnt3a-treated PK-1 cells. Wnt3a significantly increased the number of PK-1 cells. (P)RR siRNA treatment prevented the Wnt3a-induced the increase in number of cells. *P < 0.05 *vs.* scrambled siRNA cells; §*P* < 0.05 *vs.* vehicle-treated cells without Wnt3a stimulation; #*P* < 0.05 *vs.* scrambled siRNA-treated cells without Wnt3a stimulation; ¶*P* < 0.05 *vs.* (P)RR siRNA cells without Wnt3a stimulation. (c) Representative xenograft formation *in vivo* of scrambled siRNA-(***left***) or (P)RR siRNA-(***right***) transfected PANC-1 cells at four weeks postinjection. (d) Average tumor volume 25 days (***left***) and 40 days (***right***) after injected with scrambled siRNA- or (P)RR siRNA-transfected PANC-1 cells (mean ± SEM, *n* = 7, *P < 0.0001).

Cell proliferative ability was evaluated by Water-soluble tetrazolium salt (WST-1) assays in three human PDAC cell lines (Fig. 4a). Treatment with Wnt3a (150 ng/mL) significantly increased the proliferative ability of PK-1 and BxPC-3 cells, with and without scrambled siRNA transfection, although Wnt3a did not significantly change the basal proliferative ability of PANC-1 cells. In all three PDAC cell lines, treatment with siRNA against (P)RR decreased (P)RR protein expression and markedly decreased cell proliferation. Even under conditions of no stimulation of Wnt3a, we also observed a significant difference in the cell proliferative ability between treatments with (P)RR siRNA and with scrambled siRNA in three different human PDAC cell lines, which inferred that knockdown of (P)RR promoted a reduction in the proliferation of human PDAC cells (Supplementary Fig. S3). Two-way ANOVA analysis revealed the effect of siRNA treatment to become more significant over time in PK-1 cells (Fig. 4b). Wnt3a significantly increased the number of living PK-1 cells with time, which was not affected by scrambled siRNA transfection. However, (P)RR siRNA treatment almost completely prevented the Wnt3a-induced increase in the number of living PK-1 cells. These data suggest that (P)RR plays a key role in the increase of Wnt3a-mediated PDAC cell proliferative ability.

To examine the effects of (P)RR silencing on pancreatic tumor growth *in vivo*, we injected PANC-1 cells expressing a scrambled siRNA or (P)RR siRNA. The 5 × 10^5^ cells were injected subcutaneously into the upper right flanks and tumor growth was measured with an electric caliper (*n* = 7 animals per group). Harvested xenografts were identical histologically to the PDAC (Supplementary Fig. S4). The mean tumor volume was significantly larger in the tumors growing in mice injected with PANC-1 cells expressing scrambled siRNA compared to mice injected with PANC-1 cells expressing (P)RR siRNA (25 days post-injection: 143.1 ± 51.8 *versus* 1.3 ± 0.9 mm^3^, *P* < 0.0001; 40 days post-injection: 302.1 ± 101.8 versus 1.3 ± 1.0 mm^3^, *P* < 0.0001) (Fig. 4c, d). We successfully detected plasma s(P)RR expression in nude mice inoculated with scrambled siRNA-transfected PANC-1 cells as well as in human patients with PDAC (Supplementary Fig. S5). These results demonstrated that (P)RR actively promotes the growth of pancreatic cancer cells.

**Figure 5:**
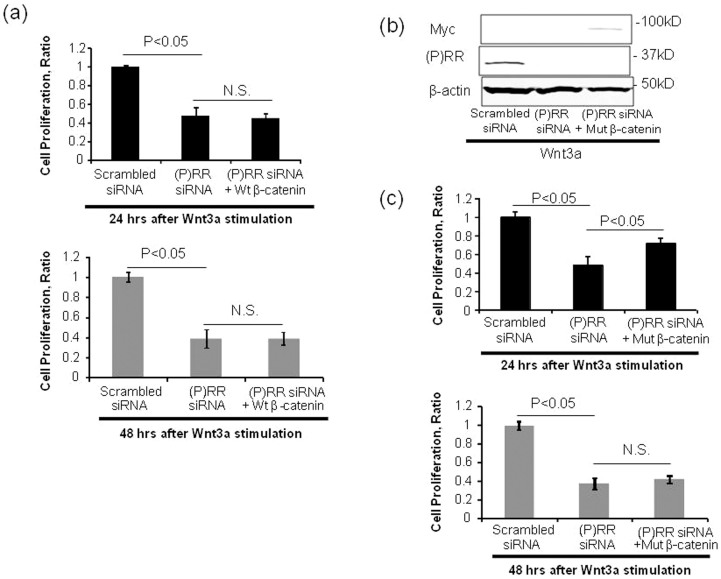
Wild-type β-catenin or constitutively active mutant β-catenin constructs were cotransfected into PK-1 cells with scrambled siRNA or (P)RR siRNA expression. (a) Effect of wild-type β-catenin (wt β-catenin) on the (P)RR siRNA-induced reduction in PK-1 cell proliferative ability. (b) The protein expression of (P)RR and Myc indicates efficiency of gene transfection with (P)RR (*ATP6AP2*) siRNA and a plasmid carrying a constitutively active aa 1–90-deleted β-catenin tagged with Myc, respectively. (c) Effect of constitutively active mutant β-catenin (mut β-catenin) on (P)RR siRNA-induced reduction in PK-1 cell proliferative ability. Cell proliferation was evaluated at 24 h and 48 h after Wnt 3a stimulation by WST-1 assays (mean ± SEM, *n* = 3 for each, *P* < 0.05 *vs.* (P)RR siRNA cells). N.S., not significant.

To investigate whether inhibition of (P)RR reduces Wnt3a-induced PDAC cell proliferation through inactivation of β-catenin, we examined the effects of co-transfection of (P)RR siRNA and a plasmid harboring wild-type β-catenin or constitutive activation of mutant β-catenin on Wnt3a-induced cell proliferative ability. Consistent with the above-mentioned data (Fig. 4a), treatment with siRNA against (P)RR significantly decreased the proliferative ability of Wnt3a-stimulated PK-1 cells (Fig. 5a–c). In these cells, overexpression of wild-type β-catenin did not affect the siRNA (P)RR-induced reduction in cell proliferative ability (Fig. 5a). Compared with cells transfected with (P)RR siRNA, constitutive activation of β-catenin significantly maintained the proliferative ability of (P)RR siRNA-transfected cells after 24 h by Wnt3a stimulation (Fig. 5b, c) but resulted in the same effect as (P)RR siRNA knockdown after 48 h (Fig. 5c). Thus, these results indicate that transfection by constitutive activation of β-catenin contributes to a delay in decreasing the proliferative ability of (P)RR siRNA-transfected cells, but the siRNA (P)RR-induced reduction in cell proliferative ability is not fully recovered through transfection with constitutive activation of β-catenin. These data confirm that (P)RR function is upstream of β-catenin in human PDAC cells, as recently demonstrated in *Xenopus* embryos^25^, but the decreased proliferative ability of (P)RR siRNA-transfected cells cannot be explained simply by inactivation of the Wnt/β-catenin signaling cascade.

### Loss of (P)RR function induces PDAC cell apoptosis

**Figure 6:**
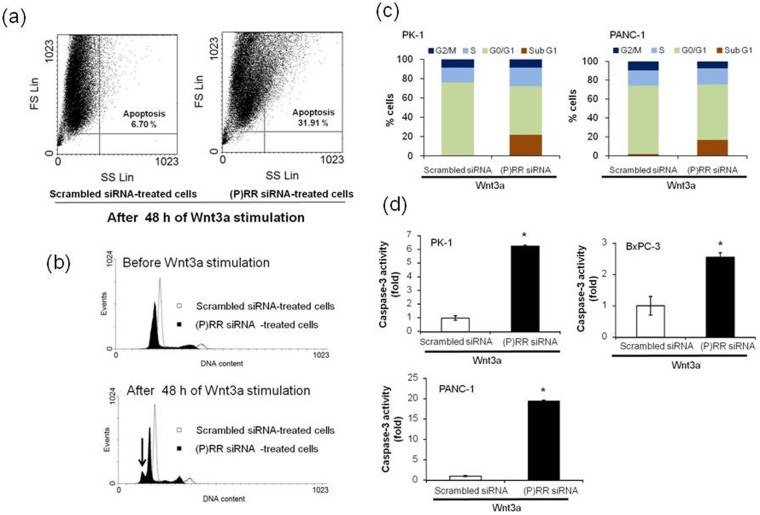
Loss of (P)RR function induces apoptosis of human PDAC cells. (a) Distributions of the populations of scrambled siRNA- and (P)RR siRNA-treated cells after 48 h of Wnt3a stimulation. Experiments were repeated in triplicate, leading to the similar results. FS: Forward Scatter; SS: Side Scatter. (b) Cell cycle analyses of scrambled siRNA- and (P)RR siRNA-treated cells stimulated with or without Wnt3a. Cells were labeled with PI and analyzed by DNA flow cytometry. The arrow shows a sub-G1 apoptotic peak. (c) Cell cycle distribution of PK-1 (***left****)* and PANC-1 (***right***) cells expressing scrambled siRNA and (P)RR siRNA after 48 h of Wnt3a stimulation. (d) Effect of (P)RR siRNA on caspase-3 activity in PK-1, BxPC-3 and PANC-1 cell lines after 48 h of Wnt3a stimulation (mean ± SEM, *n* = 3 for each, *P < 0.05 *vs.* scrambled siRNA cells). (P)RR siRNA dramatically increased caspase-3 activity in all three cell lines.

To investigate the mechanism by which (P)RR siRNA suppresses PDAC cell proliferation, DNA content was measured by PI in PK-1 cells, with detection of cells undergoing late-stage apoptosis^33^. The percentage of apoptotic cells determined by flow cytometry was higher in the (P)RR siRNA-treated case than in scrambled siRNA-treated cells after 48 h of Wnt3a stimulation (Fig. 6a). As shown by the analysis of cell cycle progression, DNA content was already reduced throughout all phases of the cell cycle in (P)RR siRNA-treated cells before Wnt3a stimulation (Fig. 6b). We detected a sub-G1 apoptotic peak in PK-1 cells treated with (P)RR siRNA, indicating that (P)RR knockdown resulted in substantial DNA decrease (Fig. 6b). Furthermore, even after stimulation with Wnt3a, the number of cells with (P)RR knocked down was increased in the sub-G1 phase in PK-1 and PANC-1 cell lines, as compared with scrambled siRNA-treated cells (Fig. 6c). However, the number of (P)RR knockdown cells was correspondingly decreased in the G0/G1 phase, when biosynthetic activity is increased before DNA replication is initiated. Taken together, these results demonstrated that the loss of (P)RR did not directly result in a decrease in cell proliferation, because G1 progression was completely prevented before the beginning of DNA replication, but the loss of (P)RR actively induced DNA lesions to trigger apoptosis in human PDAC cells. This conclusion was also supported by the observation that (P)RR siRNA-treated cells had significantly reduced cell proliferation compared with scrambled siRNA-treated cells without Wnt3a stimulation (Supplementary Fig. S3).

Cell apoptosis was also confirmed by measuring caspase-3 activity. (P)RR siRNA treatment of cells followed by Wnt3a stimulation significantly increased caspase-3 activity in all three human PDAC cells (Fig. 6d). These data showed that the loss of (P)RR resulted in apoptosis through promotion of caspase-3 activity in human PDAC cells. The possible mechanisms responsible for (P)RR in the activation of the Wnt/β-catenin signaling pathway and the progression of PDAC are depicted in Supplementary Fig. S6.

## Discussion

The present study revealed new critical aspects that strongly indicate a potential role of (P)RR in Wnt/β-catenin signaling-dependent genesis of PDAC. To our knowledge, this is the first demonstration that (P)RR might be profoundly involved in PDAC development. First, aberrant expression of (P)RR in premalignant PanIN and PDAC lesions as well as elevated plasma levels of s(P)RR was observed in patients with PDAC. Second, cultured human PDAC cell lines revealed substantial increases in full-length (P)RR expression and secretion of s(P)RR. Third, (P)RR was found to be crucial for Wnt/β-catenin-dependent proliferation of PDAC cells. Finally, loss of (P)RR triggered apoptosis of human PDAC cells through the activation of caspase-3.

Inappropriate activation of the Wnt/β-catenin-dependent signaling pathway due to mutations in genes encoding regulatory proteins of this cascade, including *CTNNB1*, *APC* and *LRP5*, has been noted in several cancers^20^. However, Wnt/β-catenin signaling-related gene mutations are rare in PDAC^11^,^15^,^16^,^17^,^18^,^19^, although constitutive activation of β-catenin occurs in PDAC cells^13^. Recently, exome sequencing and copy number analysis performed in a prospective clinical cohort of 99 patients with resected PDAC showed that mutations of *SLIT2* and *ROBO2*, which enhance β-catenin complex formation, were present in only 5% of cases^15^. Thus, activation of the Wnt/β-catenin signaling pathway cannot be solely explained by related gene mutations in PDAC. In the present study, we demonstrated that (P)RR is abundantly expressed in nearly 100% of patients, using PDAC tissues and PDAC cell lines. Wnt3a stimulation promoted increased levels of active β-catenin as soon as phosphorylation of LRP6 was initiated in PK-1 cells. Increased active β-catenin expression induced by Wnt3a stimulation is also consistent with the results obtained from a recent study that elucidated the mechanism by which Wnt controls β-catenin dynamics^34^. Furthermore, (P)RR siRNA treatment significantly attenuated LRP6 phosphorylation. It has been demonstrated that (P)RR is part of the LRP6 complex by coimmunoprecipitation from lysates of HEK293T cells transfected with different deletion mutants^25^. Our evidence supports the data by Cruciat et al. (2010). We also established that treatment with recombinant human Wnt3a enhanced expression of active β-catenin and cyclin D1, which are key components of the Wnt/β-catenin signaling pathway in PK-1 and BxPC-3 cells. (P)RR siRNA treatment resulted in a remarkable reduction in the expression of these proteins in human PDAC cells. Wnt3a stimulated the proliferation of PK-1 and BxPC-3 cells but not PANC-1 cells, which is likely owing to their high levels of basal Wnt 3a. Similar to these data, autocrine activation of Wnt/β-catenin signaling has already been reported in PDAC^14^,^18^. Interestingly, gene knockdown of (P)RR prevented Wnt3a-induced proliferation of all PDAC cell lines and dramatically decreased the proliferation of PANC-1 cells *in vivo*. Furthermore, transfection of a plasmid causing constitutive activation of β-catenin significantly attenuated siRNA (P)RR-induced reduction of cell proliferative ability, although this proved to be temporary. These data indicate that one mechanism of activation of the Wnt/β-catenin signaling pathway in PDAC is by virtue of inappropriate activation of (P)RR in the absence of mutations in genes encoding regulatory proteins of the Wnt/β-catenin signaling cascade.

Accumulation of β-catenin in the nucleus promotes cell proliferation through activation of Wnt target genes, such as *CCND1*, which increases transition through G0/G1^5^. In the present study, cyclin D1 expression was reduced following (P)RR siRNA knockdown, and attenuation of PDAC cell proliferation by (P)RR inhibition was accompanied by a substantial decrease in G0/G1 populations and an increase in the sub-G1 phase, indicating that (P)RR plays an important role in PDAC cell proliferation and inhibition of apoptosis. These observations are consistent with those of a previous study demonstrating that inhibition of β-catenin attenuates PDAC cell proliferation and induces apoptosis^13^, which also indicates that a strong connection occurs between (P)RR and β-catenin in human PDAC cells. According to results obtained from flow cytometry, (P)RR siRNA treatment not only detected the peak of sub-G1 phase but also the decrease in the total DNA content. The reason why (P)RR siRNA treatment induced the decrease of the total DNA content remains to be determined. Future studies are required to clarify these molecular mechanisms.

In the present study, transfection of (P)RR siRNA triggered apoptosis, resulting in decrease of the proliferative ability of human PDAC cells. Studies in tissue-specific (P)RR knock-out mice showed that specific deletion of (P)RR in cardiomyocytes or glomerular podocytes resulted in death a few weeks after birth^24^,^35^,^36^. Loss of (P)RR led to apoptosis of both normal and cancer cells, indicating that an appropriate (P)RR level is indispensable for cell survival. Notably, transfection by constitutive activation of β-catenin temporally enabled (P)RR siRNA-transfected human PDAC cells to maintain their proliferative ability. However, cell proliferative ability was not fully recovered, and cells eventually followed the same fate as those transfected with (P)RR siRNA alone. This is also supported by the differences in phenotypes of conditional mouse models of (P)RR and the components of Wnt/β-catenin signaling pathway. Although the longevity of the conditional mouse model for (P)RR is limited, as described above, the conditional mouse model for the components of Wnt signal transduction has a lifespan sufficiently long to enable elucidation of the role of Wnt signaling during the later stages of development and adult life^37^, which shows that the decrease in (P)RR function results in much more severe biological effects in mice. These findings suggest the involvement of (P)RR in other signaling pathways governing cell survival and proliferation. Further studies will be needed to unravel the orchestrated interactions between (P)RR and candidate signaling pathways besides the Wnt/β-catenin signaling pathway.

PanIN is a histologically well-defined precursor to invasive PDAC^28^, associated with active *KRAS* mutations and differential expression of other genes^15^,^28^. Al-Aynati *et al*. reported aberrant nuclear localization of β-catenin expression in high-grade PanIN (PanIN-2 and -3) and PDAC lesions^8^, as also demonstrated in the present study (Supplementary Fig. 2b), and Zhang *et al*.^14^ obtained evidence that activation of the Wnt/β-catenin pathway is required for PanIN formation. Of interest, the present immunohistochemical study showed that (P)RR expression was barely detectable in normal pancreatic ducts, but aberrant expression of (P)RR was evident from early stages of pancreatic carcinogenesis, i.e., PanIN-2 and PanIN-3 lesions, as well as PDAC, suggesting that inappropriate augmentation of (P)RR is essential for the survival and proliferation from early stages of pancreatic carcinogenesis through the canonical Wnt signaling pathway. Recently, epigenetic silencing of *DKK* and *SFRP* genes, which encode inhibitors of the receptor-ligand binding at the top of the Wnt signaling cascade, was established as another mechanism known to driving Wnt signaling in cancers including PDAC^38^. There is little doubt that the Wnt/β-catenin signaling pathway is a key pathway in multiple cancers and is disrupted though a variety of mechanisms. Although inappropriate activation of (P)RR might be one mechanism of activation of Wnt/β-catenin signaling, our data offer the attractive possibility of developing a new class of drugs in cancer therapy and repositioning known drugs.

The present study provided another novel insight into PDAC. Although data supporting the essential role of the Wnt/β-catenin signaling pathway in pancreatic carcinogenesis are accumulating as stated above, specific biomarkers of Wnt/β-catenin activation are lacking. One of the major problems is that no components of the Wnt/β-catenin signaling pathway have been detected in serum or plasma to date^1^. In the present study, we measured s(P)RR with a commercially available ELISA kit^39^ and demonstrated that plasma levels in patients with PDAC were significantly higher than those in healthy matched controls, suggesting that plasma s(P)RR could be a potential biomarker for PDAC. Further studies involving a larger number of patients are warranted to establish diagnostic sensitivity and specificity of s(P)RR in plasma.

## Methods

### Human plasma and tissue samples

The institutional committee for ethics of Kagawa University and the National Cancer Center approved our studies using human plasma and tissue samples and informed consent was obtained from all subjects before the study was started. Eligible subjects were defined in advance as 20 patients with PDAC who were admitted to the National Cancer Center Hospital, Tokyo, Japan, between July 2012 and August 2012. The plasma of healthy volunteer examinees was collected at the Research Center for Cancer Prevention and Screening, a branch of the National Cancer Center, during the same period (between July 2012 and August 2012). To prevent sampling biases, 20 frequency-matched volunteer examinees in eight age categories (50–54, 55–59, 60–64, 65–69, 70–74, 75–79, 80–84 and 85–89 years of age) and matched sex were chosen as potential healthy controls. No cancer was detected by cancer screening at the institution in any of these 20 volunteer examinees.

Patients with PDAC and healthy volunteer examinees were scheduled for blood collection before any procedures on the first day of admission or screening, respectively. Fasting venous blood was drawn into a vacutainer tube with EDTA-2Na. All 20 patients with PDAC and all 20 healthy volunteer examinees had fasted. The blood samples were centrifuged to obtain blood plasma and these specimens were preserved at −80°C until analysis.

### Human s(P)RR assays by sandwich ELISA

Human s(P)RR assays were performed in duplicate with use of a commercial kit (Immuno-Biological Laboratories, Gunma, Japan, catalog #27782). Recombinant human (P)RR was employed as the standard. The human (P)RR standard (125–8,000 pg/mL diluted in ELISA buffer) and plasma (1:9 dilution in ELISA buffer) samples (100 μL/well) were individually loaded into wells of 96-well plates, which were incubated at 4°C overnight. Then, the plates were washed a total of five times with washing buffer (PBS containing 0.05% Tween 20). After incubation with horseradish peroxidase-labeled anti-human renin receptor antibodies (100 μL/well, 1:30 dilution in solution) at 4°C for 1 h, the plates were washed a total of five times with washing buffer. They were incubated with 3,3′,5,5′-tetramethylbenzidine solution (100 μL/well) under light-protected conditions at room temperature for 45 min. The reaction was stopped by the addition of sulfuric acid (100 μL/well, 0.5 mol/L) and absorbance was measured at 450 nm with a plate reader.

### Immunohistochemistry of (P)RR and β-catenin in PDAC tissues

Immunohistochemistry of (P)RR was performed using surgical specimens of PDAC. Briefly, paraffin sections were deparaffinized and incubated with methanol containing 0.3% hydrogen peroxide for 15 min, then 10% normal goat serum (Nichirei Biosciences, Tokyo, Japan, catalog #426042) was added to the sections to block non-specific staining. The sections were incubated with an anti-human (P)RR rabbit polyclonal antibody^40^,^41^ or an anti-human β-catenin monoclonal antibody (1:1000 dilution, BD Transduction Laboratories, San Jose, CA, catalog #610154) for 1 h at room temperature. After washing with PBS, sections were also incubated with the secondary antibody (rabbit IgG conjugated with horseradish peroxidase, Nichirei Biosciences, catalog #424151) for 40 min at room temperature. Sections were visualized by immersion in DAB (3,3′ diaminobenzidine, Nichirei Biosciences, catalog #415172) as a chromogen. Then nuclear staining was performed using hematoxylin, and each section was embedded in malinol. (P)RR and β-catenin immunolabeling was evaluated by Y.S. and S.Y. Negative controls were included using non-immune serum instead of the primary antibody.

### Cell culture and conditioned medium

Dr. T. Furukawa (Institute for Integrated Medical Sciences, Tokyo Women's Medical University) kindly supplied BxPC-3, PCI-35 and PK-8 cells for us. PANC-1 and MIAPaCa-2 cells were purchased directly from the American Type Culture Collection (ATCC; Manassas, VA, USA). PK-1 cell was obtained from Tohoku University. BxPC-3, PCI-35, PK-8, PK-1, MIAPaCa-2 and PANC-1 were grown in RPMI-1640 media (Sigma-Aldrich, St. Louis, MO, catalog #R8758) supplemented with 10% fetal bovine serum (Nichirei Biosciences, Tokyo, Japan, catalog #17012), penicillin (50 U/mL) and streptomycin (50 μg/mL, Life Technologies, Carlsbad, CA, catalog #15070). HPDE cells were cultured in Hu-Media KG2 containing 10 mg/mL insulin, 0.1 μg/mL human EGF, 0.5 mg/mL hydrocortisone hemisuccinate, 50 mg/mL gentamycin, 50 μg/mL amphotericin B and 0.4% V/V BPE (Kurabo, Osaka, Japan, catalog #KK-2150S). According to the cell culture method recommended by ATCC, hTERT-HPNE (CRL-4023) were grown in one volume of Medium M3 Base (Incell Corp., San Antonio, TX, catalog #M300F-500) and three volumes of DMEM without glucose (Sigma-Aldrich, St. Louis, MO, catalog #D5030 with 2 mM L-glutamine and 1.5 g/L sodium bicarbonate), 5% FBS, 5.5 mM D-glucose, 10 ng/mL human recombinant EGF, and 750 ng/mL puromycin. These cell lines were maintained at 37°C under 5% CO_2_/95% air in a humidified incubator. Serum-free conditioned medium was obtained by starving each cell line for 24 h. Conditioned medium was concentrated with an Amicon Ultra Device (10 kDa cut-off, Millipore, Billerica, MA, catalog #UFC501024). Cells were lysed with 100 μL of ice-cold lysis buffer, pH 7.4 (in mM: 50 HEPES; 5 EDTA; 100 NaCl), 1% Triton X-100, protease inhibitors (10 μg/mL aprotinin, 1 mM phenylmethylsulfonyl fluoride, 10 μg/mL leupeptin), and phosphatase inhibitors (in mM: 50 sodium fluoride; 1 sodium orthovanadate; 10 sodium pyrophosphate, 0.001 microcystin) and the supernatant was obtained after centrifugation for 10 min at 12,500 rpm.

### Transient gene transfection by siRNA and plasmid DNA

Cells were treated with 150 ng/mL recombinant human Wnt3a (R&D systems, Minneapolis, MN, catalog #5036-WNP-010), and harvested for analyses. For transient gene transfection, BxPC-3, PANC-1 and PK-1 cells were serum starved for 24 h and then transfected with (P)RR (*ATP6AP2*) siRNA and a plasmid carrying constitutively active aa 1–90-deleted β-catenin tagged with Myc or a full-length β-catenin cDNA before stimulation by Wnt3a. Stealth Select RNAi (catalog #HSS115476), a predesigned (P)RR siRNA, was synthesized by Life Technologies. Plasmid carrying constitutively active β-catenin was obtained from Addgene, Cambridge, MA (catalog #31785)^42^. Plasmids carrying a full-length β-catenin cDNA were obtained from DNAFORM, Yokohama, Japan (catalog #H023067115). Based on the manufacturer's recommended protocol, each cell line was transfected with 100 pmol (P)RR siRNA using Lipofectamine RNAiMAX (Life Technologies). Stealth RNAi siRNA Negative Control (Life Technologies, catalog #12935-400) was also applied to the cells in the same manner. Co-transfection with 100 pmol (P)RR siRNA and a plasmid harboring either constitutively active β-catenin (200 ng) or full-length β-catenin (200 ng) was conducted using X-treme GENE siRNA Transfection Reagent (Roche Applied Science, Penzberg, Germany, catalog #04476093001).

### Western blot analysis

Protein extraction and measurement of protein concentrations were performed, as previously described^43^. Total protein extracts (30 μg) were electrophoretically separated using 10% SDS-polyacrylamide gels and transferred onto nitrocellulose membranes. Blots were blocked with blocking solution LI-COR (Lincoln, NE, catalog #927-40000). The primary antibodies used (1:1000 dilution in blocking solution) were anti-active-β-catenin (Millipore, Billerica, MA, catalog #05-665), Cyclin D1 (Cell Signaling Technology, Danvers, MA, catalog #2922), Wnt3a (R&D systems, catalog #MAB1324), phosphorylated LRP6 (Ser 1490, Cell Signaling Technology, catalog #2568), anti-human (P)RR rabbit polyclonal antibody^40^,^41^ and Myc-tag (Cell Signaling Technology, catalog #2276). Secondary antibodies coupled to infra-red dyes (IRDye 800 goat anti-rabbit IgG, catalog #926-32221 and IRDye 680 goat anti-mouse IgG, catalog #926-32220) were also used (1:1000 dilution in blocking solution). Protein detection was performed using an Odyssey scanner (LI-COR). Data were normalized based on the expression of targeted protein in vehicle-treated cells. To confirm equal protein loading, membranes were reprobed with an antibody against β-actin (Sigma-Aldrich, St. Louis, MO, catalog #A5441) or LRP6 (Cell Signaling Technology, catalog #3395). To obtain a loading control of s(P)RR expression, we used the CBB (Coomassie Brilliant Blue) staining solution (Wako, Osaka, Japan, catalog #17400553) to visualize the protein bands.

### *In vivo* tumorigenicity studies

Experimental protocols and animal care were performed according to the guidelines for the care and use of animals established by Kagawa University. The Animal Experimentation Ethics Committee at Kagawa University approved the experiments. Five-week-old male BALB/c nude mice (nu+/nu+) purchased from CREA, Japan were housed under pathogen-free conditions. Mice were anesthetized with sevoflurane. PANC-1 cells, 5 × 10^5^ (expressing scrambled siRNA or (P)RR siRNA), in 200 μL of PBS were injected subcutaneously into the upper right flanks using a 26G needle (*n* = 7 per group). Size of local tumors at the implanting site was measured with an electric caliper. Volumes of tumors were calculated as follows: tumor volume (mm^3^) = length × (width)^2^ × 0.5. The tumor tissue of each mouse was fixed, embedded, sectioned serially, stained with hematoxylin and eosin, and examined under a microscope.

### WST-1 assays and direct PDAC cell counting to determine cell proliferative ability

Prior to transfection, 5 × 10^4^ cells were seeded per well of 6-well plates. After siRNA-transfected cells were stimulated with Wnt3a for 24 or 48 h, WST-1 assays were performed according to the manufacturer's protocol (TAKARA BIO, Otsu, Japan, catalog #MK-400) to determine proliferative ability. One hundred microliters of WST-1 reagent was added to the cell culture medium in each well. After incubation for 2 h, the absorbency of the samples was measured with a microplate reader at a wavelength of 450 nm. Direct cell counting was performed to count the cells at 24, 48 and 72 h after Wnt3a stimulation. Then, the cells were treated with 0.25% Trypsin-EDTA (Life Technologies, catalog #25200-056), and then resuspended in PBS after centrifugation. The cell suspensions were stained with 0.5% trypan blue stain solution (Nacalai Tesque, Kyoto, Japan, catalog #29853-34) to distinguish living cells from dead cells, and 10 μL of each cell suspension was loaded into the sample injection area within C-Chip (Digital Bio, Seoul, Korea, catalog #DHC-N01). Living cell numbers per mL were calculated.

### Detection of apoptotic cells using propidium iodide (PI) DNA content and caspase-3 assays

After 48 h of Wnt3a stimulation, siRNA-transfected cells were resuspended in 0.5 mL of ice-cold 70% ethanol after centrifugation and kept at −20°C for 24 h. Following washing in PBS, cells were resuspended in 10 mg/mL RNase A (Macherey-Nagel, Düren, Germany, catalog #740505) in PBS for 30 min at 37°C and stained with 1 mg/mL PI (Sigma-Aldrich, catalog #P4170) for 30 min at 37°C. After washing with PBS, cells were resuspended in fresh PBS, and data were acquired using a DNA flow cytometer. After treating the siRNA-transfected cells with Wnt3a, caspase-3 assays were performed according to the manufacturer's protocol (Medical & Biological laboratories, Nagoya, Japan, catalog #4800) to detect caspase-3 activity. Cell lysates, 2× reaction buffer and caspase-3 substrate were added to each well and incubated for 2 h at 37°C, then the absorbency of the samples was measured with a microplate reader at a wavelength of 405 nm. The data were calculated based on the standard curve and normalized to the total protein concentration of the cell extracts.

### Statistical analysis

Results are expressed as mean ± SEM, with the exception of s(P)RR concentrations (mean ± SD). Continuous variables were compared using Student's *t* test for s(P)RR concentration in human and nude mice and tumor volume *in vivo*. We used one-way ANOVA with Scheffe's post hoc test for analyzing protein expression, Wnt3a-stimulated cell proliferative ability measured by WST-1 assays. We also performed two-way ANOVA to detect the effects of treatment by siRNA and stimulation period by Wnt3a in number of PK-1 cells. *P* < 0.05 was considered significant. All the statistical analyses were performed using IBM SPSS Statistics 20 (Armonk, NY) software.

## Author Contributions

The author(s) have made the following declarations regarding their contributions: Y.Sh., G.N., S.Y. and A.N. designed this study. Y.Sh., T.F. and S.Y. acquired the data. Y.Sh., T.F., G.N., D.N., S.Y. and A.N. performed the analysis and interpretation of data. Y.Sh., G.N., S.Y. and A.N. wrote the paper. A.I. and H.K. revised the manuscript. T.H., K.T., K.K., M.K., T.M. and Y.Su. contributed reagents/materials/analytical tools.

## Supplementary Material

Supplementary Information Supplementary information
