# Supplementary material for: (Pro)renin receptor is crucial for Wnt/β-catenin-dependent genesis of pancreatic ductal adenocarcinoma
Source: Sci Rep. 2015 Mar 9;5:8854. doi: 10.1038/srep08854 (PMC4352858; doi:10.1038/srep08854)
Supplement: Supplementary Information [file srep08854-s1.pdf]

Supplementary information

(Pro)renin receptor is crucial for Wnt/β-catenin-dependent genesis of pancreatic ductal adenocarcinoma

Yuki Shibayama[1], Takayuki Fujimori[2], Genevieve Nguyen[5], Takuo Hirose[5], Kazuhito Totsune[6], Atsuhiro Ichihara[7], Kento Kitada[1], Daisuke Nakano[1], Hiroyuki Kobori[1], Masakazu Kohno[3], Tsutomu Masaki[2], Yasuyuki Suzuki[4], Shinichi Yachida[4,8] and Akira Nishiyama[1]

Departments of [1]Pharmacology, [2]Gastroenterology and Neurology, [3]Cardiorenal and Cerebrovascular Medicine, and [4]Gastroenterological Surgery, Faculty of Medicine, Kagawa University, Kagawa 761-0793, Japan

[5]Center for Interdisciplinary Research in Biology (CIRB), Collège de France, Centre National de la Recherche Scientifique (CNRS) Unités Mixtes de Recherche (UMR) 7241, Institut National de la Santé et de la Recherche Médicale (INSERM) U1050, Paris 75014, France.

[6]Department of Planning for Drug Development and Clinical Evaluation, Tohoku University Graduate School of Pharmaceutical Science and Medicine, Sendai 980-8577, Japan.

[7]Department of Hypertension and Endocrinology, Tokyo Women's Medical University Tokyo 162-8666, Japan.

[8]Division of Refractory Cancer Research, National Cancer Center Research Institute, Tokyo 104-0045, Japan

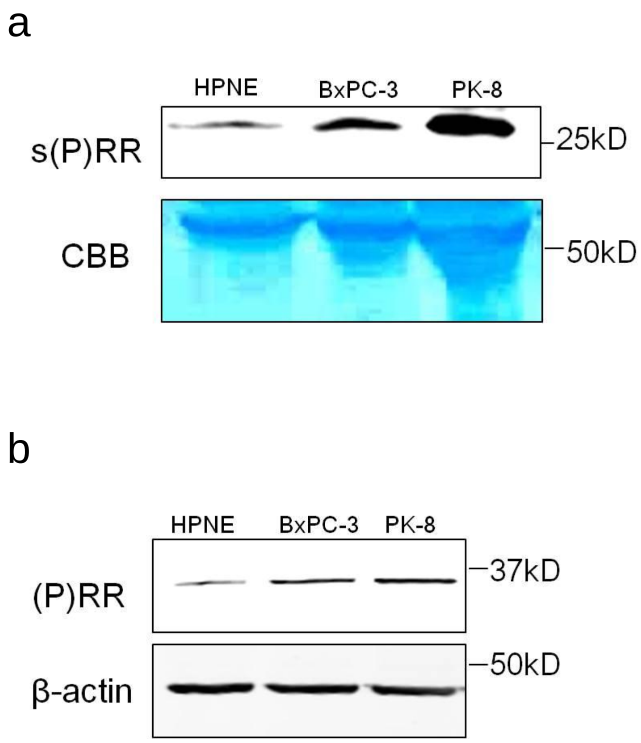

Supplementary Figure S1 (P)RR expression was assayed for HPNE and two different PDAC cells. (a) S(P)RR expression in conditioned medium. Control for loading was determined by CBB staining. **(b)**Full-length (P)RR expression in cell lysates. β-actin was used as loading control. Consistent results were observed when three experiments were repeated.

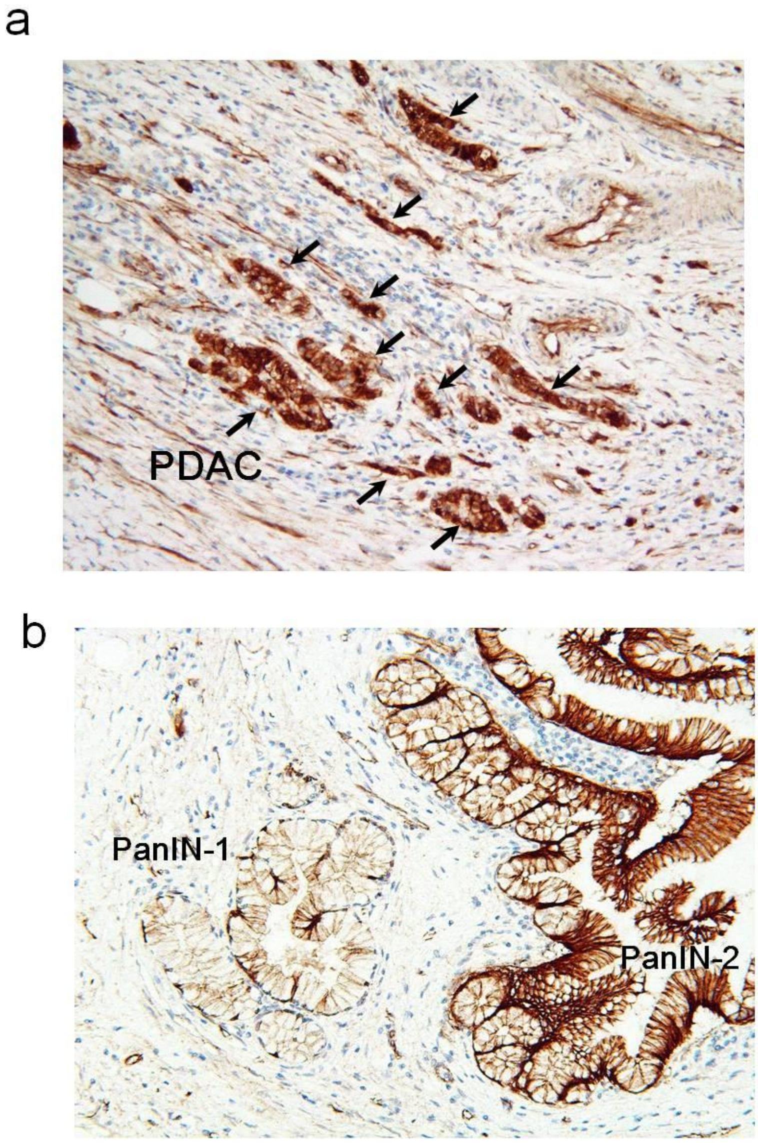

Supplementary Figure S2 Typical immunohistochemical labeling profiles of β-catenin in pancreatic ductal adenocarcinoma (PDAC) tissues. (**a**) PDAC cells (*arrows*) show strong β-catenin immunoreactivity in both the cytoplasm and nuclei. (**b**) β-catenin expression in PanIN-1 and PanIN-2 lesions in representative pancreatic tissue samples. The PanIN-2 lesions show strong β-catenin immunoreactivity in the cytoplasm and/or nuclei, although PanIN-1 lesions show only faint β-catenin expression in the cell membranes.

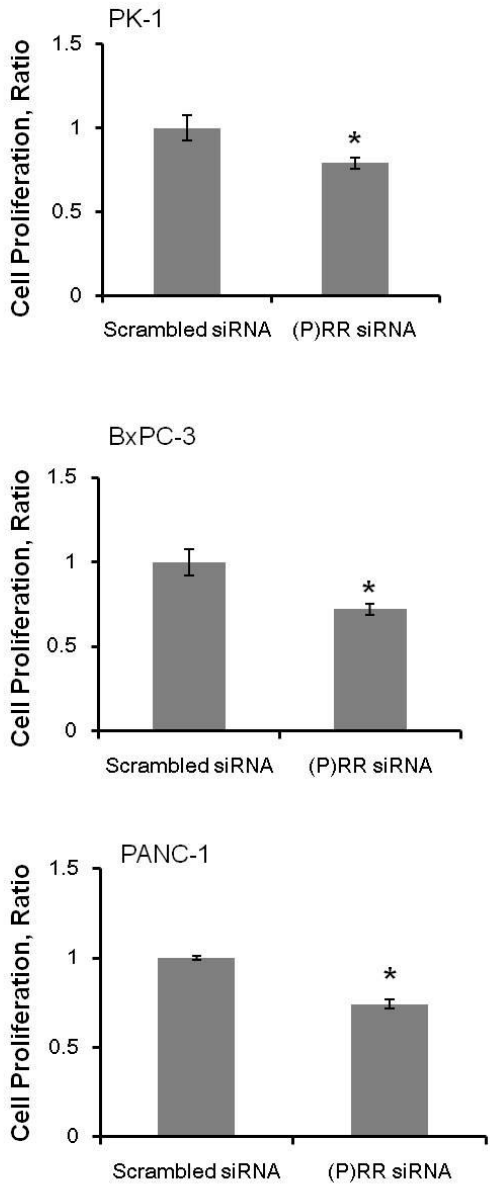

Supplementary Figure S3 Effect of (P)RR siRNA transfection on proliferation of human PDAC cells without Wnt3a stimulation, as evaluated by WST-1 assays. Compared with the scrambled siRNA-treated cells, cell proliferation was significantly reduced by (P)RR siRNA (Mean ± SEM, n = 3 for each, *$P < 0.05$).

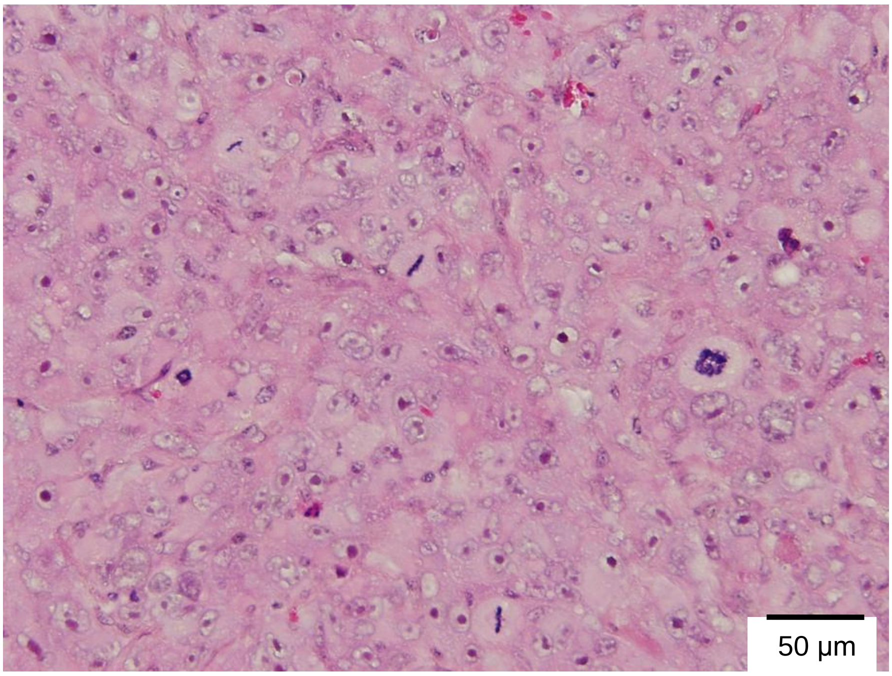

Supplementary Figure S4 Histology of heterotransplanted tumor tissue in a nude mouse inoculated with scrambled siRNA-transfected PANC-1 cells. Abundant mitoses are also evident (hematoxylin and eosin stains).

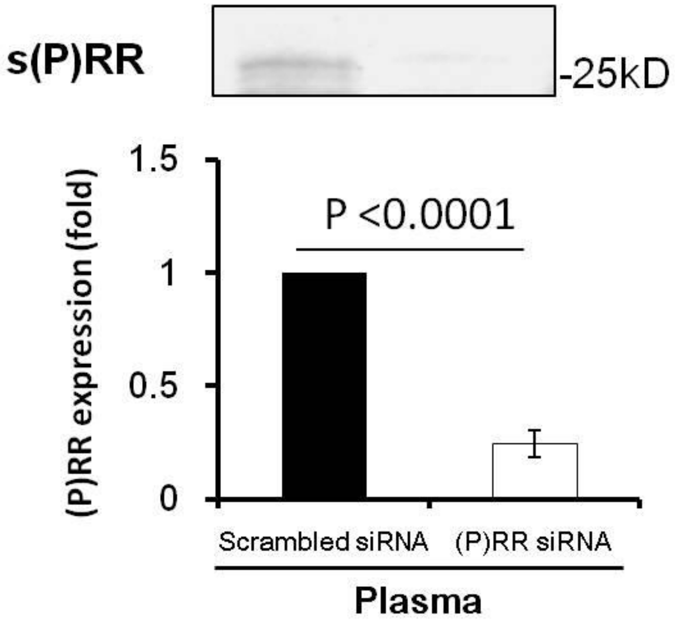

Supplementary Figure S5 Plasma s (P)RR expression in mice injected with scrambled siRNA- or(P)RR siRNA-transfected PANC-1 cells. Plasma (P)RR expression in nude mice inoculated with scrambled siRNA-transfected PANC-1 cells was successfully detected as well as in human patients with PDAC (Mean ± SEM, n = 5 for each, P < 0.0001 *vs.* (P)RR siRNA cells).

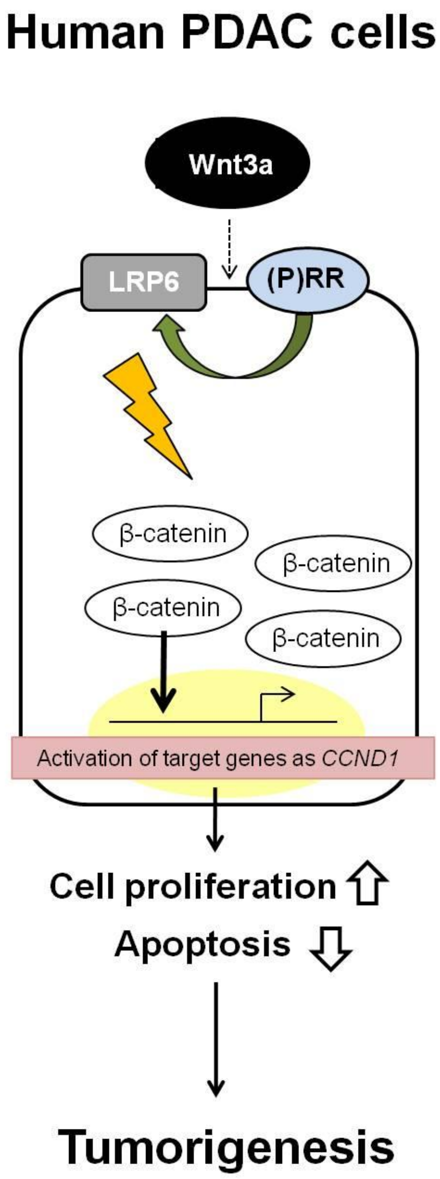

Supplementary Figure S6 Schematic diagram summarizing the potential role of (P)RR in the activation of the Wnt/β-catenin signaling pathway and the progression of pancreatic ductal adenocarcinoma (PDAC). (P)RR plays a key role in mediating Wnt3a-induced β-catenin activation in human PDAC cells. Aberrant (P)RR expression activates Wnt/β-catenin signaling, which inhibits apoptosis and promotes proliferation of PDAC cells.

Supplementary Table S1 Age and gender of 20 health control subjects for measuring plasma soluble (P)RR concentration.

Patient No.	Age	Gender
1	74	F
2	55	M
3	77	M
4	52	M
5	54	M
6	66	M
7	80	F
8	64	M
9	76	M
10	61	F
11	54	M
12	54	M
13	59	M
14	48	M
15	56	M
16	64	M
17	68	F
18	74	M
19	60	F
20	64	M

Abbreviations: M, Male; F, Female.

Supplementary Table S2 Clinical characteristics of 20 patients with pancreatic ductal adenocarcinoma for measuring plasma soluble (P)RR concentration.

**Patient No.**	**Age**	**Gender**	**T (UICC)**	**N (UICC)**	**M (UICC)**	**Stage (UICC)**
1	57	M	x	x	1	IVB
2	67	M	x	x	1	IVB
3	74	M	4	x	0	III
4	54	M	3	1	0	II
5	79	M	x	x	1	IVB
6	64	F	3	1	0	II
7	55	M	4	x	0	III
8	67	F	4	x	0	III
9	60	M	x	x	1	IVB
10	53	M	x	x	1	IVB
11	46	M	4	x	0	III
12	86	F	4	x	0	III
13	52	M	x	x	1	IVB
14	73	F	4	x	0	III
15	71	M	4	x	0	III
16	53	M	x	x	1	IVB
17	57	M	x	x	1	IVB
18	63	F	3	0	0	III
19	62	M	x	x	1	IVB
20	62	M	4	1	0	IVA

Abbreviations: M, Male; F, Female; UICC, Union for International Cancer Control.

Supplementary Table S3 Clinicopathologic features of 22 patients with pancreatic ductal adenocarcinoma undergoing pancreatic resection.

Patient No.	Age	Sex	T (UICC)	N (UICC)	M (UICC)	Stage (UICC)	Tumor size (mm)	Tumor differentiation	IHC of (P)RR
1	72	F	2	1	0	II	25	Grade 2	+
2	77	F	3	0	0	II	40	Grade 1	+
3	85	F	3	0	0	II	NA	Grade 3	+
4	74	F	2	0	0	I	40	Grade 1	+
5	65	F	3	1	0	II	40	Grade 2	+
6	68	M	3	1	0	II	25	Grade 2	−
7	69	M	3	1	0	II	20	Grade 2	+
8	67	M	3	0	0	II	37	Grade 1	+
9	75	M	3	0	0	II	40	Grade 3	+
10	76	F	3	1	0	II	25	Grade 1	+
11	63	M	3	1	0	II	28	Grade 2	+
12	81	F	3	1	0	II	60	Grade 2	+
13	65	M	3	0	0	II	35	Grade 1	+
14	63	F	3	1	0	II	55	Grade 1	+
15	77	M	3	1	0	II	30	Grade 1	+
16	78	M	3	1	0	II	28	Grade 3	+
17	73	M	3	0	0	II	40	Grade 1	+
18	65	M	3	0	0	II	41	Grade 2	+
19	73	M	3	0	0	II	39	Grade 1	+
21	47	F	3	1	0	II	42	Grade 1	+
22	72	F	3	1	0	II	30	Grade 1	+

Abbreviations: M, Male; F, Female; UICC, Union for International Cancer Control; NA, not available; IHC, immunohistochemistry.
